# Influence of iTBS on the Acute Neuroplastic Change After BCI Training

**DOI:** 10.3389/fncel.2021.653487

**Published:** 2021-03-12

**Authors:** Qian Ding, Tuo Lin, Manfeng Wu, Wenqing Yang, Wanqi Li, Yinghua Jing, Xiaoqing Ren, Yulai Gong, Guangqing Xu, Yue Lan

**Affiliations:** ^1^Department of Rehabilitation Medicine, Guangzhou First People's Hospital, School of Medicine, South China University of Technology, Guangzhou, China; ^2^Sichuan Provincial Rehabilitation Hospital, Chengdu University of Traditional Chinese Medicine, Chengdu, China; ^3^Department of Rehabilitation Medicine, Guangdong Provincial People's Hospital, Guangdong Academy of Medical Sciences, Guangzhou, China

**Keywords:** transcranial magnetic stimulation, brain computer interface, functional near-infrared spectroscopy, intermittent theta burst stimulation, motor imagery

## Abstract

**Objective:** Brain-computer interface (BCI) training is becoming increasingly popular in neurorehabilitation. However, around one third subjects have difficulties in controlling BCI devices effectively, which limits the application of BCI training. Furthermore, the effectiveness of BCI training is not satisfactory in stroke rehabilitation. Intermittent theta burst stimulation (iTBS) is a powerful neural modulatory approach with strong facilitatory effects. Here, we investigated whether iTBS would improve BCI accuracy and boost the neuroplastic changes induced by BCI training.

**Methods:** Eight right-handed healthy subjects (four males, age: 20–24) participated in this two-session study (BCI-only session and iTBS+BCI session in random order). Neuroplastic changes were measured by functional near-infrared spectroscopy (fNIRS) and single-pulse transcranial magnetic stimulation (TMS). In BCI-only session, fNIRS was measured at baseline and immediately after BCI training. In iTBS+BCI session, BCI training was followed by iTBS delivered on the right primary motor cortex (M1). Single-pulse TMS was measured at baseline and immediately after iTBS. fNIRS was measured at baseline, immediately after iTBS, and immediately after BCI training. Paired-sample *t*-tests were used to compare amplitudes of motor-evoked potentials, cortical silent period duration, oxygenated hemoglobin (HbO2) concentration and functional connectivity across time points, and BCI accuracy between sessions.

**Results:** No significant difference in BCI accuracy was detected between sessions (*p* > 0.05). In BCI-only session, functional connectivity matrices between motor cortex and prefrontal cortex were significantly increased after BCI training (*p*'s < 0.05). In iTBS+BCI session, amplitudes of motor-evoked potentials were significantly increased after iTBS (*p*'s < 0.05), but no change in HbO2 concentration or functional connectivity was observed throughout the whole session (*p*'s > 0.05).

**Conclusions:** To our knowledge, this is the first study that investigated how iTBS targeted on M1 influences BCI accuracy and the acute neuroplastic changes after BCI training. Our results revealed that iTBS targeted on M1 did not influence BCI accuracy or facilitate the neuroplastic changes after BCI training. Therefore, M1 might not be an effective stimulation target of iTBS for the purpose of improving BCI accuracy or facilitate its effectiveness; other brain regions (i.e., prefrontal cortex) are needed to be further investigated as potentially effective stimulation targets.

## Introduction

Brain computer interface (BCI) can directly translate brain activities reflecting the subject's intention into motor commands for controlling an external device (Abiri et al., 2019). The external device can in turn provide BCI users with state-dependent sensory feedback, which is known as closed-loop BCI system (Johnson et al., 2018). Following stroke, the connection between the peripheral muscles and sensorimotor cortex is often disrupted due to cortical or subcortical lesions, which results in hemiparesis. With BCI, stroke survivors are able to control external devices bypassing the damaged physiological motor output system (Daly and Wolpaw, 2008), including those with severe hemiparesis who cannot actively participate in traditional motor training (Ramos-Murguialday et al., 2013). The closed-loop BCI system provides stroke survivors with a chance to actively participate in motor training and activate their motor-related cortices (Pichiorri et al., 2015). The effectiveness of BCI training on motor recovery following stroke has been reported in several clinical studies (Teo and Chew, 2014; Pichiorri et al., 2015; Sun et al., 2017; Wu et al., 2019).

However, a large portion of BCI users (~30% of healthy adults and ~40% stroke survivors) are unable to control BCI systems effectively (Blankertz et al., 2010). This phenomenon is called “BCI-illiteracy” problem (Vidaurre and Blankertz, 2010), which largely limits the effectiveness of BCI training. “BCI-illiteracy” has been suggested to result from insufficient event-related desynchronization (ERD) of the mu rhythm (Buch et al., 2008). Mu rhythm is typically observed over the sensorimotor area with a frequency of 8–13 Hz and is attenuated during motor imagery (i.e., mu ERD). As the amplitude of mu ERD plays a crucial role in BCI decoding accuracy (Buch et al., 2008), it is sometimes difficult for BCI systems to detect the subject's motion intention without a strong mu ERD (Kasashima et al., 2012). As the amplitude of mu ERD is related to motor-related cortical activation, it can be modulated by non-invasive brain stimulation (NIBS) techniques (Hummel and Cohen, 2006). Therefore, NIBS is a feasible approach for enhancing BCI performance and solving “BCI-illiteracy” problem. The influence of transcranial direct current stimulation (tDCS), a common form of NIBS, on BCI training has been extensively investigated (Ang et al., 2015; Kasashima-Shindo et al., 2015; Hong et al., 2017). It has been reported that anodal tDCS applied on motor cortex can increase mu ERD and improve BCI accuracy in both healthy adults (Wei et al., 2013) and stroke survivors (Ang et al., 2015; Kasashima-Shindo et al., 2015). Unfortunately, the increased BCI accuracy cannot be transferred to improved effectiveness of BCI training in motor recovery following stroke (Ang et al., 2015; Kasashima-Shindo et al., 2015; Hong et al., 2017); this is possibly because anodal tDCS could not facilitate neuroplastic changes induced by BCI training. Therefore, how other forms of NIBS influence the effects of BCI training needs to be investigated.

Repetitive transcranial magnetic stimulation (rTMS) is another popular NIBS technique, which is frequently applied to induce modulation of cortical activation. Among patterned rTMS protocols, a modified form of rTMS known as theta-burst stimulation (TBS) can induce longer-lasting neural effects with shorter application time and lower stimulation intensities compared with conventional rTMS paradigms. Intermittent TBS (iTBS) has been suggested to have facilitatory neural effects (Chung et al., 2016). When applied on the primary motor cortex (M1), robust increase in cortical excitability usually lasts for 20–30 min after iTBS (Huang et al., 2005, 2011; Chung et al., 2016). Based on its convenience of use and strong neural modulatory effect, iTBS has been widely applied as a powerful technique to upregulate cortical excitability in clinical studies (Chung et al., 2016; Chen et al., 2019). Therefore, iTBS might be an effective approach for increasing BCI accuracy and facilitating neuroplastic changes of BCI training.

Neuroplastic changes after BCI training can be investigated by many neuroimaging approaches, such as functional magnetic resonance imaging (fMRI), electroencephalography (EEG), and functional near-infrared spectroscopy (fNIRS), etc. (Yang et al., 2019). Although being able to provide accurate information about brain structure and neural activities, high cost and subject's head fixation have limited applications of fMRI in tasks that require constant movement or real-time monitor (Strangman et al., 2006; Mihara and Miyai, 2016). fNIRS is a non-invasive neuroimaging tool that monitors cerebral and myocardial oxygenation during tasks (Yang et al., 2019). Compared with fMRI, fNIRS has some advantages including real-time monitor, relatively low price, simplicity, and relatively high temporal resolution. Compared with EEG, fNIRS has higher spatial resolution and is less likely to be influenced by subject's head move during motor tasks (Yang et al., 2019). Therefore, fNIRS is a suitable approach for monitoring immediate neuroplastic changes after BCI training.

Here, we investigated how iTBS targeted on M1 influences the BCI accuracy and acute neuroplastic changes of BCI training. We used fNIRS to measure acute neuroplastic changes of BCI training. We anticipated that BCI training would increase brain activation and functional connectivity in the motor and prefrontal cortices. iTBS would improve BCI accuracy and boost the acute neuroplastic changes induced by BCI.

## Methods

### Participants

Eight healthy adults volunteered for this two-session study [four males; mean age: 21.6 (SD = 1.2) years]. Participants were included only if they were right-handed assessed with the Edinburgh Handedness Inventory, and had no history of neurological disorders, including no head or hand injuries. Participants were excluded if: using medications that reduce seizure threshold; pregnant; or any implanted device or metal that might be affected by the magnetic field generated by TMS was present.

Participants gave their written informed consent for the experimental procedures that were approved by the Guangzhou First People's Hospital Human Research Ethics Committee. The study was performed in accordance with the Declaration of Helsinki.

### Force Measurements

We tested maximal voluntary isometric pinch grip (MVC) in both hands of each participant. Grip force assessment system (BioFlex-H, Zhanghe Intelligent Co., Guangzhou, China) was used to measure isometric pinch grip force in the “standard” position (Ding and Patten, 2018) with real-time force feedback displayed on a 24-inch television screen. Three MVC trials were interspersed with rest intervals (2 min); the peak value was carried forward as MVC for each hand.

### Physiological Measures

#### Transcranial Magnetic Stimulation

Surface electromyography (EMG) was recorded from the first dorsal interosseus (FDI) of both hands. Participants were seated in a comfortable chair with back supported. The EMG raw signal was amplified and band-pass filtered (3 Hz−3 kHz), digitized at a sampling rate of 2,048 Hz with a 50 Hz notch filter enabled. EMG data were written to disc for offline analysis.

TMS was performed using a NS5000 Magnetic Stimulator (YIRUIDE Medical Co., Wuhan, China). TMS was applied over M1 using a figure-of-eight-shaped coil (70 mm diameter) positioned tangentially 45° from midline to induce a posterior-anterior current in the hemisphere. Participants were asked to remain static while determining the optimal scalp position for eliciting maximal responses in the contralateral FDI. Resting motor threshold (RMT) was determined experimentally as the lowest stimulation intensity that produced motor evoked potentials (MEP) ≥ 50 uV in 50% of consecutive stimulations at rest (Chen et al., 1998). Active motor threshold (AMT) was determined experimentally as the lowest stimulation intensity that produced MEPs ≥ 200 uV in 50% of consecutive stimulations during grip at 10% MVC (Matsunaga et al., 2005; Takechi et al., 2014). A neuronavigation system (Visor2, ANT Neuro, Hengelo, Netherlands) was used to ensure reliable and consistent coil positioning over the hotspot throughout the experiment. Coil position error was controlled at <5 mm displacement and <3° relative to the target (Ding et al., 2018). Stimulations were delivered at every 5–8 s. Both hemispheres were tested in a random order.

Totally 40 single-pulse TMS pulses were delivered at 120% RMT, with 20 stimuli at rest and 20 during grip at 10% MVC. At rest condition, participants were instructed to completely relax. EMG signals displayed on a computer screen were used to provide feedback and assist participants in keeping the arm and hand muscles quiet. During grip, participants produced constant submaximal (10% MVC) isometric pinch grip with force feedback displayed visually as a target zone (10 ± 2% MVC) within which the participant was instructed to maintain force. Prior to the testing, participants practiced using visual feedback to maintain the force trace within the target zone. TMS was applied when the force trace was stable and maintained in the target zone.

MEPs were analyzed offline using custom written Matlab scripts (Matlab2019b, Mathworks, Inc., Natick, USA). EMG data were demeaned, filtered using a fourth order Butterworth filter (10–500 Hz), and signal averaged over 20 trials for each condition. Averaged peak-to-peak MEP amplitudes of both rest and grip condition were calculated (i.e., MEP_rest_ and MEP_active_, respectively). Cortical silent period (CSP) was also calculated. CSP onset was defined as the point at which the average rectified EMG amplitude remained below threshold for 5 ms. CSP offset was defined as the point at which the amplitude returned to and remained above threshold for 5 ms. The CSP duration was quantified by the time interval between CSP onset and offset (Triggs et al., 1992; Classen et al., 1997; Urbin et al., 2015).

#### Functional Near-Infrared Spectroscopy

**Figure 1:**
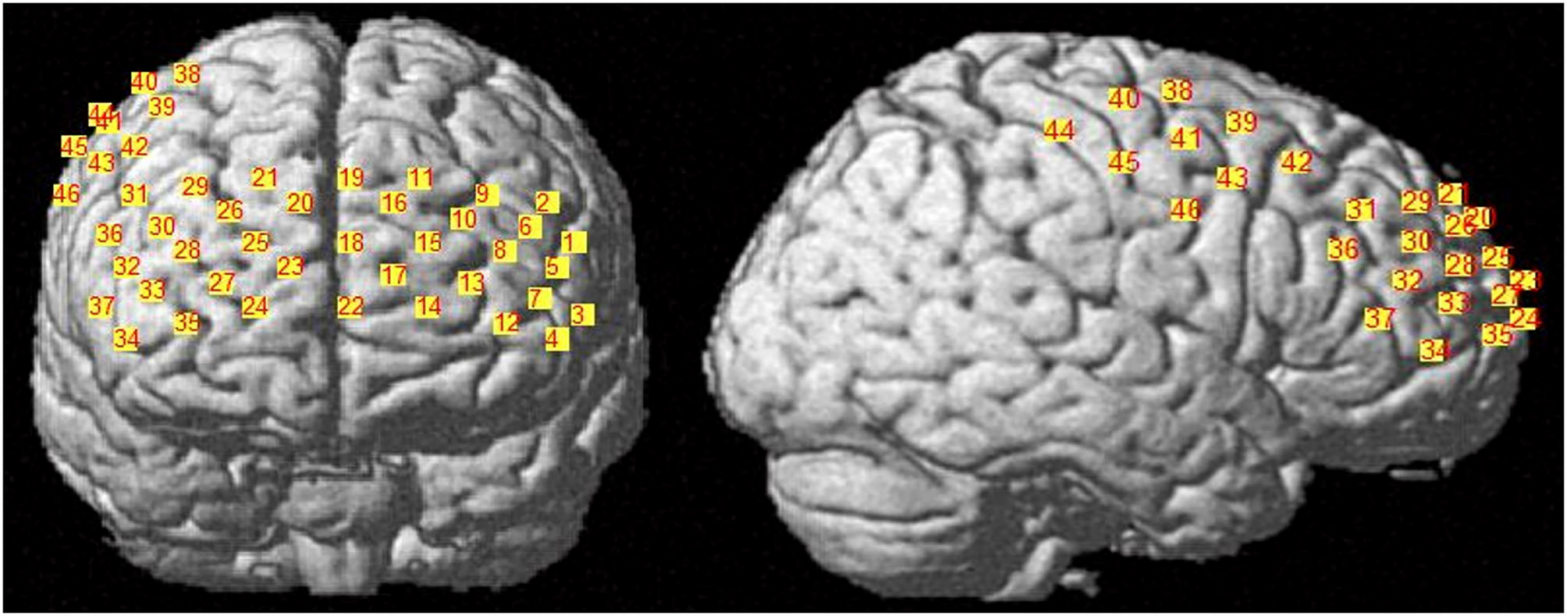
fNIRS 46-channel montage placement. There were 37 channels placed on the prefrontal cortex, and eight channels placed on the right motor cortex.

fNIRS data was acquired using a 46 multichannel fNIRS instrument (BS-3000, Wuhan Znion Technology Co., Wuhan, China). Channels between each transmitter and receiver were placed with reference to the 10–20 system. The two probe sets were inserted into a nylon cap and then placed on the participant's head. One of the probe sets was placed on the prefrontal area (24 optodes, 37 channels), and the other one was placed on the right motor cortex (8 optodes, 9 channels). The 46-channel montage placement is shown in Figure 1. A chin strap was used to secure the cap in place to reduce cap movement. Prior to recording, a NIR gain quality check was performed to ensure data acquisition was neither under-gained nor over-gained. Data were recorded at wavelengths of 695 and 830 nm.

**Figure 2:**
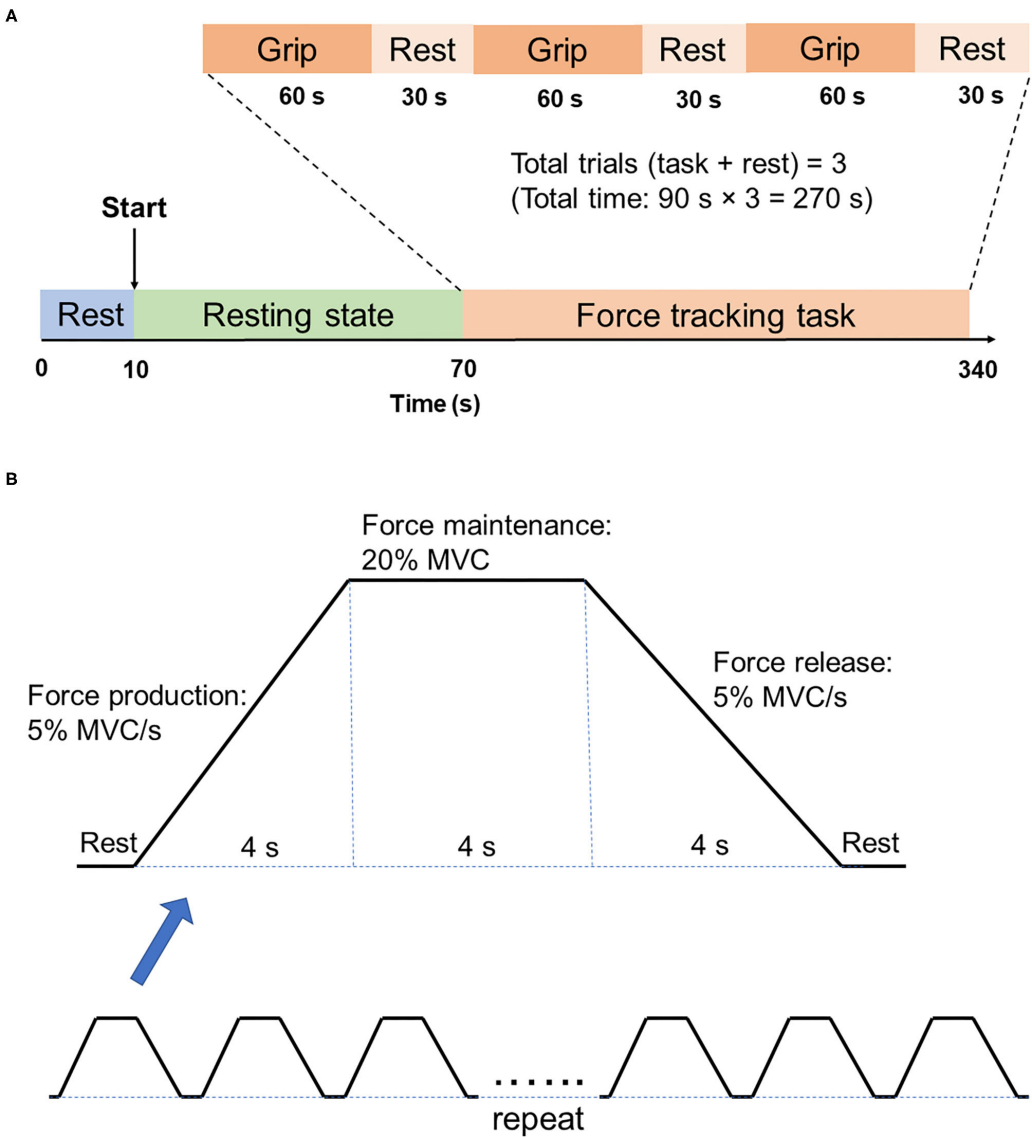
Illustration of fNIRS testing. **(A)** fNIRS testing procedure. There are two states of fNIRS testing, which are the 60s resting state and 270s force tracking task. Force tracking task consists of three trials, with each trial followed by 30s rest. **(B)** Force tracking task paradigm. Each block of force tracking task includes force production phase, force maintenance phase and force release phase, with 4s rest break between two adjacent blocks.

For each fNIRS testing, participants were asked to rest for 60 s, followed by the force tracking task (60 s grip and 30 s rest for 3 times) (Figure 2A). At rest condition, participants were asked to sit in an armchair with eyes closed. During the force tracking task, subjects were asked to use a pinch grip to produce submaximal isometric contraction to track the criterion trajectory (Figure 2B) as accurately as possible. The force tracking task included force production, force maintenance and force release phase.

Regions of interest (ROIs) were selected *via* Polhemus PATRIOT digitizer channel registration analyses. After tasks were completed, subjects were instructed to keep the fNIRS cap on while the experimenters carefully removed the optodes. A measuring tape was used to find the center point (i.e., Cz) on the head. Measurements were taken from the left auricular lobule to the right auricular lobule, and from the nasion to the inion. Once the Cz point was determined, a magnet was positioned on it and the subject was moved so that the inion was 10 cm away from the transmitter. Five head base reference points were measured using the stylus, which are nasion, left tragus, right tragus, inion, and Cz. All other optical fiber points were measured in numerical order afterwards. Selected ROIs were M1, left and right dorsal lateral prefrontal cortex (DLPFC), left and right frontal polar (FP). All channels with >50% overlap within a region were averaged together based on MRIcro registration (Rorden and Brett, 2000; Wan et al., 2018).

Fluctuations in concentration of delta oxygenated hemoglobin (HbO2) were calculated from changes in detected light intensity according to the modified Beer-Lambert Law, assuming constant scattering (Sakatani et al., 2006). Data preprocessing was performed after delta HbO2 signals were obtained. We used the moving average filter was 3 s (Huo et al., 2019). A processing method based on moving standard deviation and cubic spline interpolation was then applied to remove motion artifacts (Scholkmann et al., 2010). Artifacts were distinguished by identifying the sliding window standard deviation above a certain threshold and were removed by cubic spline interpolation. The physiologic signals are removed from the data using the low pass filer with a cut-off of 0.2 Hz. The low-frequency drift was removed by a high pass filter of 0.01 Hz cut-off frequency (Arun et al., 2020). 60s task period and 10s rest period before the trial were extracted from the data. Baseline correction was then performed on each trial to ensure that the beginning of each task period was approximately zero. The average time series for each ROI (i.e., hemodynamic response function, HRF) was calculated for the force tracking task. The averaged amplitude of each HRF was calculated for statistical analysis.

Functional connectivity was calculated in both time and frequency domain. In the time domain, correlation approach was used to estimate the strength of the pairwise Pearson's correlation between ROIs (Pannunzi et al., 2017). In frequency domain, coherence and phase locking value (PLV) were used to analyze the level of synchronization of the fNIRS signals. The Welch's averaged, modified periodogram method (Welch, 1967), was performed to calculate the squared coherence between ROIs. PLV was calculated to indicate the stability of the phase difference between two time series [for calculation details see Briels et al. (2020)]. All connectivity matrices were Fisher's *z*-transformed (Arun et al., 2020) to the set of Gaussian distributed values and the *z* scores were used for further statistical analysis.

### Interventions

#### BCI Training

**Figure 3:**
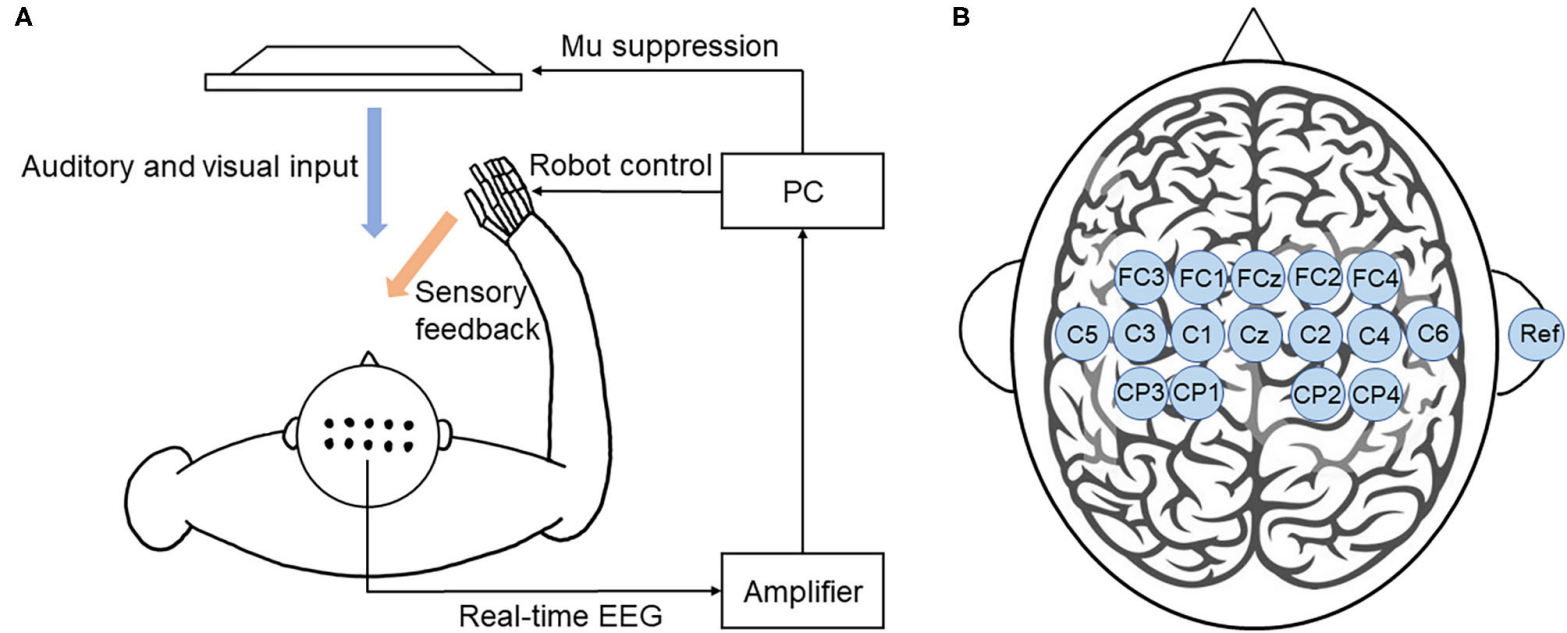
BCI training system. **(A)** Illustration of the closed-loop system. This BCI system consists of an EEG amplifier collecting real-time EEG, a PC analyzing EEG signal and providing visual and auditory feedback, and a robot hand providing sensory feedback. **(B)** The location of EEG electrodes. There were 16 active electrodes placed on the motor and sensory area and a reference electrode placed on the right ear.

Motor imagery-based BCI training system was developed as shown in Figure 3A. EEG signals were recorded using 16 active electrodes (g.LADYbird, g.Tec Medical Engineering GmbH, Schiedlberg, Austria). The real-time EEG signals were amplified (g.USBamp, g.Tec Medical Engineering GmbH, Austria) and then computer processed. Video clips were played in a 24-inch computer screen to guide the participants to complete motor imagery tasks. An exoskeleton hand was used to provide sensory feedback in hand grasping/opening tasks. A mu ERD score (0–100) was displayed on the screen to provide real-time feedback. Subjects could adjust their motor imagery strategy according to this index to achieve higher scores and control the BCI robot more effectively (Sun et al., 2017).

EEG signals were referenced to a unilateral earlobe. The signal from 16 active electrodes was sampled at 256 Hz. The real-time EEG signals were processed by the amplifier using a band-pass filter (2–60 Hz) to remove artifacts and a notch filter (48–52 Hz) to remove power line interference. All electrodes were filled with salt water to ensure the transmission impedance remained below 1 kOhm. The EEG electrodes were placed over the central area according to the 10–20 system (Figure 3B). EEG signals from the C3 and C4 electrodes were used for BCI control.

During the action observation, a dark screen was first displayed for 2 s, followed by a white cross for 2 s. Then, a text cue of “hand grasp” or “hand open” was displayed for 2 s. A video clip with a duration of 6 s was then displayed. Subjects were asked to observe the actions and imagine they were performing those actions without actually moving their hands. The mu ERD score was calculated based on the EEG signal during the video. If the mu ERD score was above 60, the robot hand would assist the subject in completing the hand grasp/open task during the following 3 s, which was considered as a successful trial. If the mu ERD score was below 60, the robot hand would not move, which was considered as an unsuccessful trial. The mu ERD score was then shown for 2 s. Each trial ended with the display of a dark screen for 3 s. During each BCI training, the trial repeated for 100 times and video clips of the grasping/opening hand was shown alternately at a random order. There was 30 s rest break after every 10 trials. Each BCI training took about 40–50 min in total. The BCI accuracy was then calculated by the number of successful trials divided by the number of total trials.

#### Intermittent Theta Burst Stimulation

iTBS was applied over the right M1 and was delivered using a NS5000 Magnetic Stimulator (YIRUIDE Medical Co., Wuhan, China). The TBS pattern consist of bursts containing three pulses at 50 Hz repeated at 5 Hz and an intensity of 80% AMT. A 2 s train of TBS was repeated every 10 s for a total of 190 s (600 pulses) (Huang et al., 2005). Participants were asked to stay relaxed during the application of iTBS.

### Experimental Procedures

**Figure 4:**
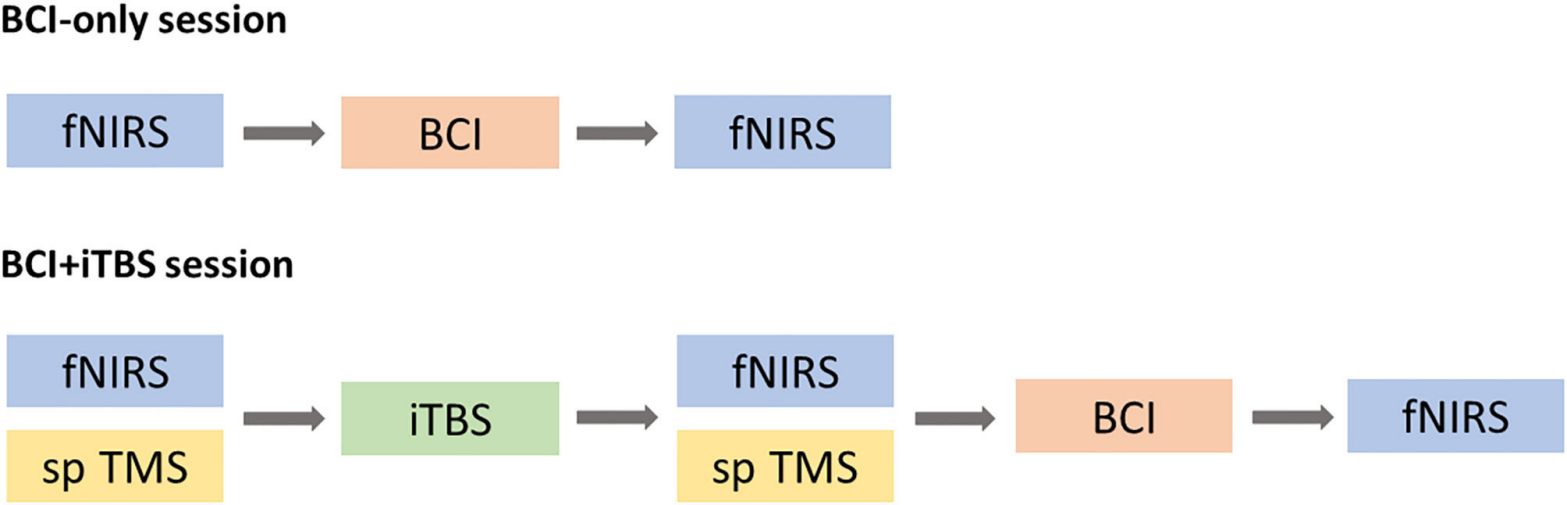
Experimental procedure. In BCI-only session, fNIRS was tested at baseline and after BCI training (top panel). In iTBS + BCI session, fNIRS was tested at three times points: baseline, after TBS and after BCI. Single-pulse (sp) TMS was tested at baseline and after iTBS. The order of those two sessions was randomized.

This study included a BCI-only session and iTBS+BCI session in a random order with approximately 7 days apart. The order of two sessions was determined by the random integers generated in Matlab2019b (Mathworks, Inc., Natick, USA). For the odd numbers, BCI-only session would be the first session and iTBS+BCI session would be the second session. For the even numbers, iTBS+BCI session would be the first session and BCI-only session would be the second session. In BCI-only session, fNIRS was tested at baseline and immediately after BCI tasks. In iTBS+BCI session, BCI training were followed by iTBS. Single-pulse TMS was tested at baseline and immediately after iTBS. fNIRS was tested at baseline, immediately after iTBS, and immediately after BCI training (Figure 4).

### Statistical Analysis

All data analyses and statistics were performed in Matlab2019b (Mathworks, Inc., Natick, USA). Data were tested using the Kolmogorov-Smirnov test and found to be normally distributed. In the BCI-only session, paired-sample *t-*tests were used to compare averaged HbO2 amplitude and functional connectivity matrices (including correlation, coherence and PLV) in each ROI before and after BCI. In the iTBS+BCI session, repeated-measures ANOVA were used to compare averaged HbO2 amplitude and functional connectivity matrices at baseline, after TBS and after BCI. In the iTBS+BCI session, paired t-test was used to compare TMS measures (including MEP_rest_, MEP_active_ and CSP) before and after iTBS. Paired-sample *t*-test was also used to compare the averaged mu ERD score during BCI training between two sessions. False discovery rate corrections were used for multiple comparisons. Statistical significance was established at *p* < 0.05.

## Results

All eight participants completed two sessions of experiment, with five participants completed BCI-only session first. No adverse effect was reported.

### BCI Accuracy

The mean BCI accuracy was 82.63% (SD = 3.6) and 81.50% (SD = 3.0) in the BCI-only session and iTBS+BCI session, respectively. There was no difference in BCI accuracy between two sessions (*p* > 0.05).

### TMS Measures

**Figure 5:**
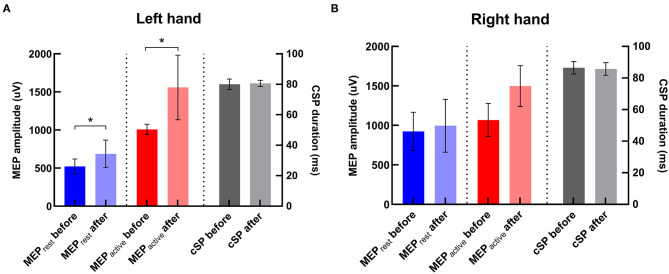
Changes in TMS parameters after iTBS in two hemispheres. **(A)** In the left hand (i.e., the contralateral hand to iTBS), MEP_rest_ and MEP_active_ were significantly increased compared with baseline, and there was no significant change in CSP duration after iTBS. **(B)** In the right hand, there was no significant change in any TMS parameter after iTBS. *Indicates significant changes after iTBS.

There was significant increase in amplitudes of MEP_rest_ and MEP_active_ in the contralateral hand (i.e., left hand) after iTBS (*p*'s = 0.02 and 0.04, respectively) (Figure 5). No significant change in CSP duration was revealed in the contralateral hand after iTBS (*p* > 0.05). There was also no significant change in MEP amplitude or CSP duration in the ipsilateral hand (i.e., right hand) after iTBS (*p*'s > 0.05).

### HRF

**Figure 6:**
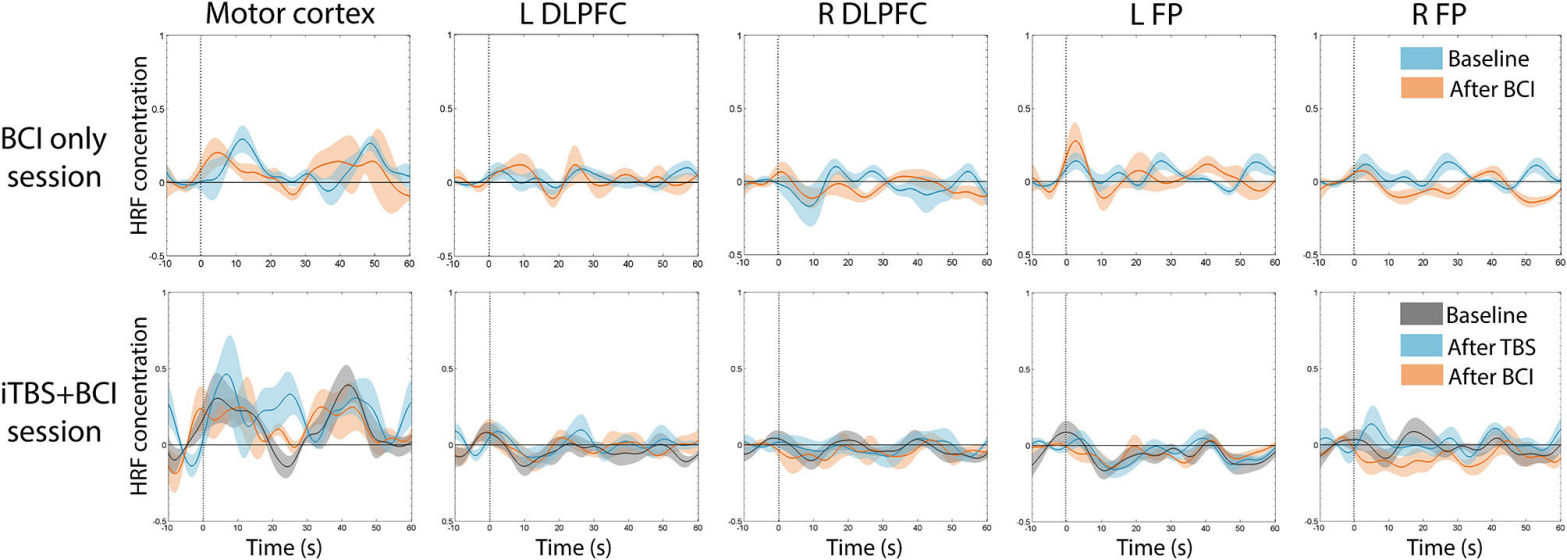
Average time series of HRF across participants. There was no significant change in average HbO2 amplitude at any time point in either session. In BCI-only session (top panel), blue indicates HRF at baseline, and pink indicates HRF after BCI training. In iTBS+BCI session (bottom panel), gray indicates HRF at baseline; blue indicates HRF after TBS; and pink indicates HRF after BCI training. Error bars are standard error.

There was no significant difference in averaged amplitude of HbO2 concentration change throughout the whole experiment in either session (Figure 6).

### Functional Connectivity Analysis

**Figure 7:**
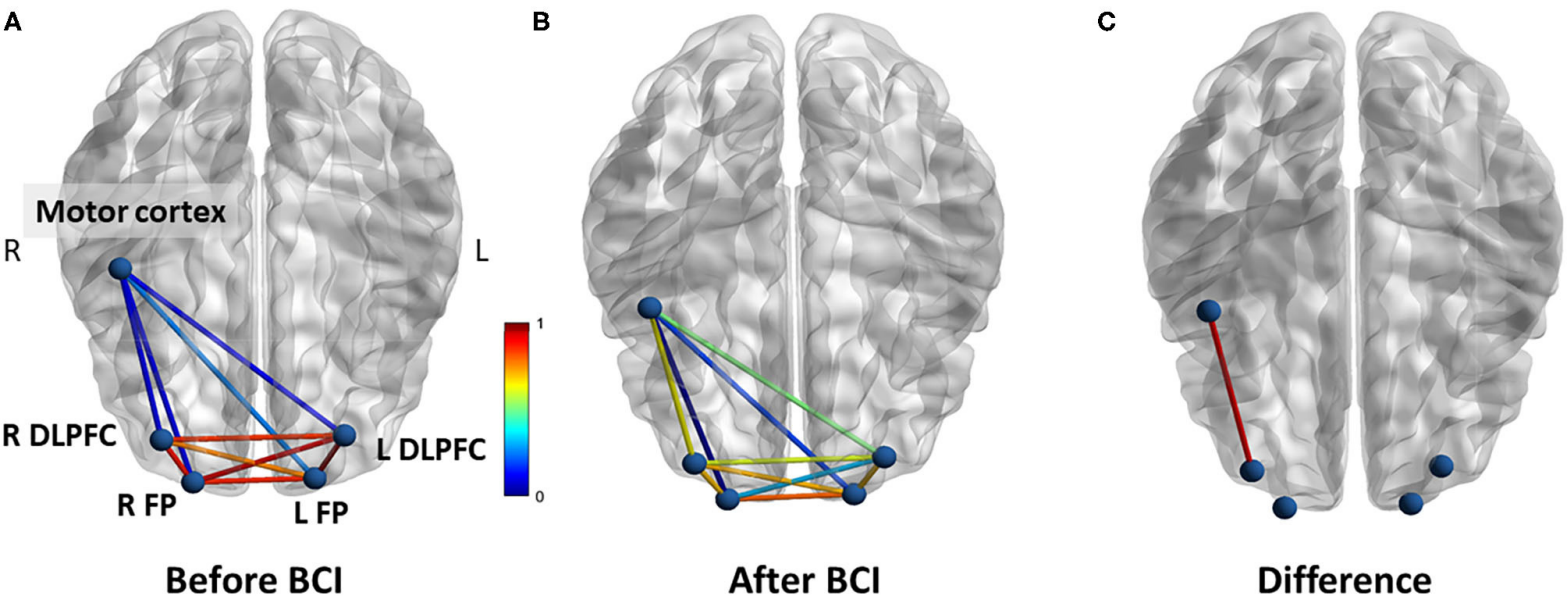
Functional connectivity (correlation) change measured at rest in BCI-only session. **(A)** Correlations between each ROI at baseline. **(B)** Correlations between each ROI after BCI training. **(C)** The correlation between right motor cortex and right DLPFC was significantly increased after BCI training. L refers to left. R refers to right. DLPFC refers to dorsal lateral prefrontal cortex. FP refers to frontal polar area.

For resting-state functional connectivity, increased correlation between motor cortex and right DLPFC was observed after BCI training in the BCI-only session (*p* = 0.005) (Figure 7). No significant difference in resting-state functional connectivity was observed throughout the iTBS+BCI session (*p*'s > 0.05).

**Figure 8:**
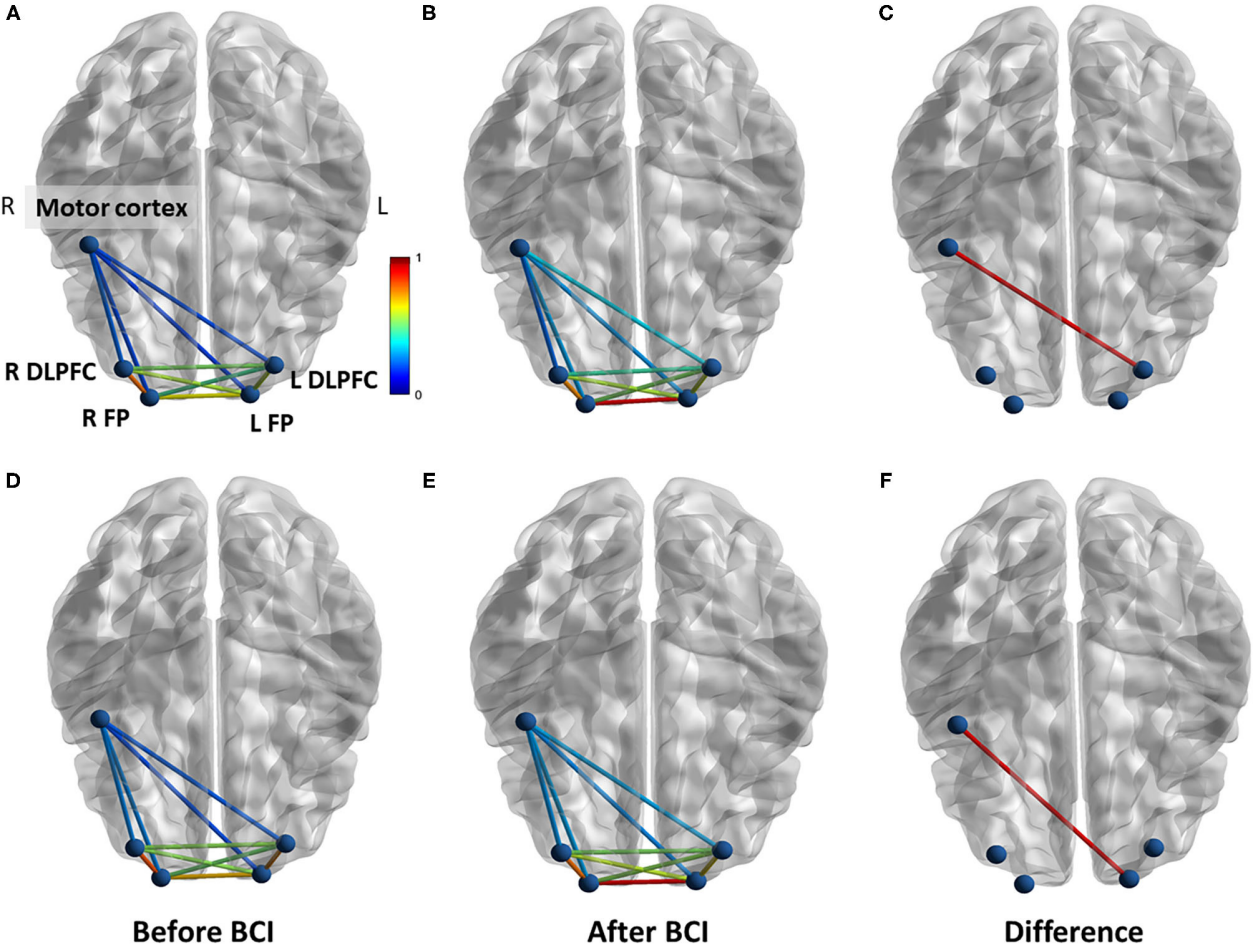
Functional connectivity change measured during force tracking task in BCI-only session. **(A)** Coherence between each ROI at baseline. **(B)** Coherence between each ROI after BCI training. **(C)** Coherence between right motor cortex and left DLPFC was significantly increased after BCI training. **(D)** PLV between each ROI at baseline. **(E)** PLV between each ROI after BCI training. **(F)** The PLV between right motor cortex and left FP was significantly increased after BCI training. L refers to left. R refers to right. DLPFC refers to dorsal lateral prefrontal cortex. FP refers to frontal polar area.

For functional connectivity measured during force tracking task, increased coherence between motor cortex and left DLPFC was observed after BCI training in the BCI-only session (*p* = 0.032) (Figure 8). In addition, increased PLV was observed between motor cortex and left FP after BCI training in the BCI-only session (*p* = 0.037). There was no significant difference in functional connectivity measured during force tracking task throughout the iTBS + BCI session.

## Discussion

This study for the first time investigated the acute neuroplastic changes after BCI training using fNIRS. We also investigated how iTBS targeted on M1 influenced BCI accuracy and neuroplastic changes induced by BCI training. Results revealed that functional connectivity between motor cortex and prefrontal cortex was acutely increased after BCI training. After iTBS, increased cortical excitability was observed, but brain activation or functional connectivity remained unchanged. iTBS targeted on M1 did not influence BCI accuracy or facilitate the neural effects induced by BCI training.

### Acute Neuroplastic Change After BCI Training

Results revealed acute neuroplastic change in functional connectivity between motor cortex and prefrontal cortex after BCI training. To our knowledge, ours is the first study that investigated the acute neural plasticity induced by BCI training using fNIRS. The acute neural adaptation of BCI training has been investigated using TMS and MRI (Xu et al., 2014; Mrachacz-Kersting et al., 2016; Nierhaus et al., 2019). TMS studies reported that MEP amplitude was increased after BCI training for up to 30 min in both stroke survivors (Mrachacz-Kersting et al., 2016) and healthy adults (Xu et al., 2014), suggesting BCI training could induce a prolonged increase in cortical excitability in the trained hemisphere (Bai et al., 2020). Nierhaus et al. (Nierhaus et al., 2019) used functional and structural MRI after only 1 h of BCI training to investigate immediate brain plasticity. Results revealed increased BOLD activity in the left sensorimotor area of trained hemisphere during motor imagery after BCI training, suggesting BCI training facilitates recruitment of cortical motor neurons during motor imagery. Our findings extend the acute neural adaptations after BCI training from cortical excitability and brain activation to functional connectivity of the cortical networks which has not been previously investigated.

As reduced functional connectivity between brain regions has been observed in stroke survivors (Arun et al., 2020), our findings suggest a potential neural mechanism underlying the effectiveness of BCI training in neurorehabilitation. Based on results from our current study and previous studies (Xu et al., 2014; Mrachacz-Kersting et al., 2016; Nierhaus et al., 2019), the neuroplastic state of motor imagery-related cortical network (including sensorimotor cortex, prefrontal cortex, etc.) is elevated after BCI training. This provides a possibility for improving the effectiveness of traditional motor training in stroke survivors by priming with BCI training, which needs to be tested in future studies.

### Acute Neuroplastic Change After iTBS

In line with previous literature (Huang et al., 2005, 2011; Cirillo et al., 2017), our results revealed increased MEP amplitudes after iTBS on M1. However, we did not observe any change in HbO2 concentration or functional connectivity in the motor cortex or prefrontal cortex after iTBS. To our knowledge, there was only one previous study using fNIRS to investigate acute neural adaptation after iTBS on M1 (Mochizuki et al., 2007). Similar to what we found in this current study, no change in HbO2 concentration in the ipsilateral motor cortex or prefrontal cortex was reported after iTBS in Mochizuki et al.'s study (Mochizuki et al., 2007).

Similar to iTBS, high-frequency rTMS is another type of non-invasive brain stimulation that has been used to upregulate cortical excitability. Some studies used fNIRS to investigate acute neural adaptation during and after high frequency rTMS on M1 (Li et al., 2017, 2019). Reduced HbO2 (Li et al., 2017) and functional connectivity (Li et al., 2019) in motor cortex and prefrontal cortex were observed in both hemispheres during rTMS, which returned to baseline after rTMS.

Collectively, there is lack of a direct relationship between hemodynamic response or functional connectivity and cortical excitability after iTBS or high-frequency rTMS. The neural mechanisms remain unclear. It has been suggested that the activation of sympathetic nervous system during rTMS application might be a possible mechanism (Li et al., 2019). The activation of sympathetic nervous system maintains a constant cerebral blood flow through vasoconstriction. This may prevent the potential dilatation of cerebral vessels induced by iTBS or high-frequency rTMS, thus no change in HbO2 concentration can be detected after stimulation (Li et al., 2019). Further studies are needed to investigate the mechanisms underlying the disassociated relationship between cortical excitability and hemodynamic response after NIBS.

### The Influence of iTBS on BCI Training

Inconsistent with our hypothesis, iTBS targeted on M1 did not influence BCI accuracy or facilitate the neuroplastic changes induced by BCI training. The mechanism is still unclear. To our knowledge, no published study has investigated the acute effect of excitatory rTMS (including iTBS) on BCI training. There were some studies investigating the influence of excitatory tDCS on BCI training (Wei et al., 2013; Ang et al., 2015; Kasashima-Shindo et al., 2015; Hong et al., 2017). It has been reported that anodal tDCS effectively increased mu ERD during BCI training and improved BCI accuracy in both healthy adults (Wei et al., 2013) and stroke survivors (Ang et al., 2015; Kasashima-Shindo et al., 2015). The different results might be due to the different stimulation paradigms. Although similar neural mechanisms are shared by iTBS and tDCS, iTBS has much higher temporal resolution compared with regular tDCS. In those aforementioned studies (Wei et al., 2013; Ang et al., 2015; Kasashima-Shindo et al., 2015; Hong et al., 2017), tDCS electrodes were relatively large and placed on motor cortex that covers not only M1 but also premotor cortex and supplementary motor cortex, while in our current study iTBS was precisely targeted on M1 with the guidance of neuronavigation system. It has been suggested that motor imagery requires not only M1 but also a distributed brain network including premotor cortex, supplementary motor cortex and prefrontal cortex, etc. (Sharma et al., 2009; Bauer et al., 2015). Because of the low spatial resolution, tDCS applied on the motor cortex might activate a broader motor imagery-related cortical network compared with iTBS targeted on M1, and thus effectively influence BCI performance. Based on this speculation, dual or multiple sites of iTBS might be more likely to improve BCI performance, which needs to be tested in the future. Difference between stroke and healthy adults may also contribute to different results between ours and previous studies (Ang et al., 2015; Kasashima-Shindo et al., 2015). As stroke survivors often have reduced mu ERD and the poorer BCI performance, there is larger room for stroke survivors to increase mu ERD or improve BCI accuracy compared with healthy adults.

Apart from BCI accuracy, iTBS on M1 did not facilitate the acute neuroplastic change induced by BCI training either. Similarly, previous studies reported that anodal tDCS on motor cortex did not facilitate neuroplastic change or improve the effectiveness of BCI training in stroke survivors (Ang et al., 2015; Kasashima-Shindo et al., 2015; Hong et al., 2017). Possibly because the use of BCI-only requires modulation of neural activities in M1 (Wander et al., 2013), M1 has been the most common target of NIBS in BCI literature (Wei et al., 2013; Ang et al., 2015; Kasashima-Shindo et al., 2015; Hong et al., 2017; Shu et al., 2017). However, acquisition of BCI proficiency requires a distributed brain network, including prefrontal cortex, premotor cortex, and posterior parietal cortex, etc. (Wander et al., 2013). In addition, the role that M1 plays in the process of motor imagery might not be as important as brain regions with higher cortical functions (e.g., prefrontal cortex) (Moghadas Tabrizi et al., 2019); this may explain why iTBS on M1 did not positively influence the effect of BCI training. Taken together, M1 may not be the best stimulation target for improving BCI accuracy or effectiveness. Further studies are needed to explore other brain regions as potentially effective stimulation targets for improving the effectiveness of BCI training.

### Limitations

As a pilot study, the sample size of current study is small (*N* = 8). In addition, our sample only includes young adults, so cautions are needed when generalizing our findings to other populations, such as aging population or stroke survivors. Future studies are needed to test our results in other populations with larger sample sizes.

Another limitation is that current study did not include sham iTBS condition in our study design. We acknowledge that the lack of sham iTBS condition may weaken the convincingness of the conclusions. In addition, due to the limited number of fNIRS channels, we did not monitor the neuroplastic changes in the left M1 (the non-stimulated hemisphere) and may miss some neuroplastic changes. Further studies are still needed to include sham iTBS condition and measure neuroplastic changes in both motor cortices.

## Conclusions

Our pilot study systematically investigated how iTBS targeted on M1 influences BCI accuracy and the acute neuroplastic changes induced by BCI training. Our results revealed that iTBS targeted on M1 did not influence BCI accuracy or facilitate the neuroplastic changes induced by BCI training, suggesting that M1 might not be an effective stimulation target of iTBS for the purpose of improving BCI accuracy or facilitate the effectiveness of BCI training. Other brain regions (i.e., prefrontal cortex) are needed to be further investigated as potentially effective stimulation targets.

## Data Availability Statement

The raw data supporting the conclusions of this article will be made available by the authors, without undue reservation.

## Ethics Statement

The studies involving human participants were reviewed and approved by Guangzhou First People's Hospital Human Research Ethics Committee. The patients/participants provided their written informed consent to participate in this study.

## Author Contributions

QD, YL, and GX designed the experiment. QD, TL, MW, WY, WL, YJ, XR, and YG conducted the experiments. QD reduced and analyzed the data. QD, YL, and GX interpreted the data. QD and YL wrote the manuscript. All authors contributed to the article and approved the submitted version.

## Conflict of Interest

The authors declare that the research was conducted in the absence of any commercial or financial relationships that could be construed as a potential conflict of interest.

## Abbreviations

BCI: brain computer interface
ERD: event-related desynchronization
NIBS: non-invasive brain stimulation
tDCS: transcranial direct current stimulation
rTMS: repetitive transcranial magnetic stimulation
iTBS: intermittent theta-burst stimulation
MVC: maximal voluntary contraction
EMG: electromyography
FDI: first dorsal interosseus
M1: primary motor cortex
RMT: resting motor threshold
MEP: motor evoked potential
AMT: active motor threshold
fNIRS: functional near-infrared spectroscopy
CSP: cortical silent period
ROI: regions of interest
DLPFC: dorsal lateral prefrontal cortex
FP: frontal polar
HbO2: oxygenated hemoglobin
HRF: hemodynamic response function
PLV: phase locking value.
